# Dephosphorylation of Centrins by Protein Phosphatase 2C *α* and *β*

**DOI:** 10.1155/2009/685342

**Published:** 2009-07-06

**Authors:** Marie-Christin Thissen, Josef Krieglstein, Uwe Wolfrum, Susanne Klumpp

**Affiliations:** ^1^Institut für Pharmazeutische und Medizinische Chemie, Westfälische Wilhelms-Universität Münster, Hittorfstr. 58-62, D-48149 Münster, Germany; ^2^Institut für Zoologie, Johannes Gutenberg-Universität Mainz, Johannes-von-Müllerweg 6, D-55099 Mainz, Germany

## Abstract

In the present study, we identified protein phosphatases dephosphorylating centrins previously phosphorylated by protein kinase CK2. The following phosphatases known to be present in the retina were tested: PP1, PP2A, PP2B, PP2C, PP5, and alkaline phosphatase. PP2C *α* and *β* were capable of dephosphorylating P-Thr^138^-centrin1 most efficiently. PP2C*δ* was inactive and the other retinal phosphatases also had much less or no effect. Similar results were observed for centrins 2 and 4. Centrin3 was not a substrate for CK2. The results suggest PP2C *α* and *β* to play a significant role in regulating the phosphorylation status of centrins in vivo.

## 1. Introduction

In the highly specialized vertebrate photoreceptor cells, centrins are components of the ciliary apparatus localized in the connecting cilium and their basal bodies [1–3]. In fully differentiated photoreceptor cells, CK2 phosphorylates centrin1 and 2 during dark adaptation. Since the phosphorylation of the ciliary centrins drastically reduces the binding to the G-protein transducin, it is suggested that the light-dependent translocation of transducin through the cilium is further regulated by CK2 phosphorylation and by the phosphatase involved.

The present study was designed to identify protein phosphatases that serve as counterparts for the CK2-mediated light-dependent phosphorylation of centrins in mammalian photoreceptor cells.

## 2. Materials and Methods

### 2.1. Phosphorylation of Centrins and BAD

GST-centrins (0.2 *μ*g) or GST-BAD (0.6 *μ*g) were incubated in 30 mM Tris-HCl, pH 7.5, 5 mM MgCl_2_, 5 mM *β*-glycerophosphate, 0.2 *μ*g CK2, 0.06% 2-mercaptoethanol, 1 mM EGTA, and 100 *μ*M ATP including 1 *μ*Ci [*γ* − ^32^P]ATP in a volume of 10 *μ*L for 15 minutes at 37°C. Then unincorporated ATP was removed by centri-SEP spin colums.

### 2.2. Dephosphorylation of P-Centrins and P-BAD

Phosphorylated proteins were incubated with 0.16 *μ*g PP1 or 0.05 *μ*g PP2A or 1.3 *μ*g PP2B or 0.08–0.8 *μ*g PP2C*α* or 0.08–1.5 *μ*g PP2C*β* or 0.08–0.8 *μ*g PP2C*δ* or 0.8 *μ*g PP5 or 1.5 *μ*g alkaline phosphatase in a total volume of 15 *μ*L, respectively. Incubations contained a 10 *μ*L aliquot of the completed phosphorylation reaction plus 5 *μ*L 50 mM Tris-HCl, pH 7.5, 1% glycerol, 0.1% 2-mercaptoethanol, and an additional 5 mM MnCl_2_ for PP1, PP2A, and PP2C*δ*; or 1 mM MgCl_2_, 0.1 mM CaCl_2_, and 2 *μ*g calmodulin for PP2B; or 1 mM MgCl_2_ for PP2C *α* and *β*; or 100 *μ*M oleic acid for PP5. Alkaline phosphatase assays contained 50 mM Tris-HCl, pH 7.9 and 1 mM MgCl_2_. Reactions were stopped after 30 minutes at 37°C by adding 5 *μ*L sample buffer (130 mM Tris-HCl, pH 6.8, 10% SDS, 10% 2-mercaptoethanol, 20% glycerol, 0.06% bromphenol blue).

## 3. Results

### 3.1. Phosphorylation of Centrins by CK2

**Figure 1:**
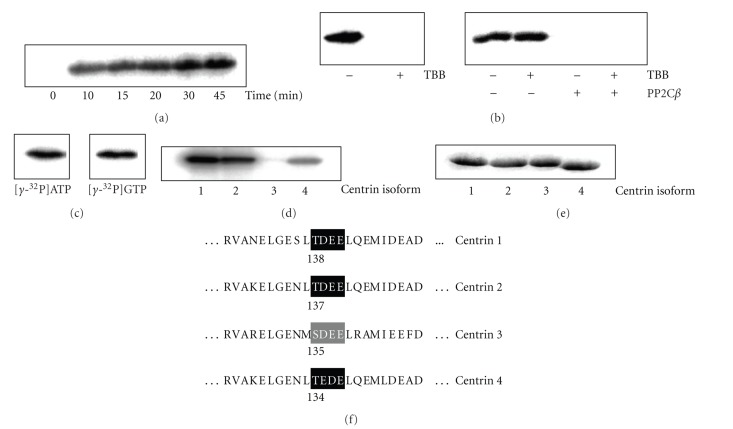
Characterization of phosphorylation of centrins by CK2. Centrins (0.2 *μ*g, resp.) were phosphorylated by CK2 (0.2 *μ*g) using [*γ* − ^32^P]ATP as phosphate source as described in Section 2. (a)–(d) Autoradiograms. (a)–(c) Centrin1 as a substrate for CK2. (a) Time dependence. (b) Effect of the CK2-inhibitor TBB (4,5,6,7-tetrabromobenzimidazole). The inhibitor was present either in the phosphorylation reaction (left) or added after phosphorylation prior to and present upon dephosphorylation by PP2C*β* (right). (c) Phosphorylation with GTP (1 *μ*Ci [*γ* − ^32^P]GTP and 100 *μ*M GTP) in comparison to that with ATP. (d) Phosphorylation of centrin isoforms (0.2 *μ*g, resp.) by CK2. (e) Coomassie protein stain of centrin isoforms (0.2 *μ*g, resp.). (f) Sequences of the CK2 phosphorylation site on the centrins 1–4.

Purified recombinant centrin1 could be phosphorylated in vitro by CK2 using ATP as phosphate source within a few minutes only (Figure 1(a)). Phosphorylation of centrin1 by CK2 was not detectable in the presence of 100 *μ*M of the CK2-inhibitor TBB (Figure 1(b)), left). Guanine nucleotides are playing a uniquely important role in the retina and for vision [3]. Indeed, phosphorylation of centrin1 by CK2 worked equally well using GTP as phosphate source instead of ATP (Figure 1(c)).

Thr^138^ of centrin1 is conserved in centrin2 (Thr^137^) and centrin4 (Thr^134^) whereas centrin3 (Ser^135^) carries a serine residue instead (Figure 1(f)). As expected from the amino acid sequence identity, centrins 2 and 4 also could be phosphorylated by CK2 (Figure 1(d)). A variety of proteins are phosphorylated by CK2 at serine residues (for review see [4]). Centrin3, however, was not a substrate of CK2 (Figure 1(d)). Coomassie staining was used in parallel to verify equal protein loading (Figure 1(e)).

### 3.2. Identification of the Phosphatases Hydrolyzing P-Centrins

Phosphatases acting on P-centrin1 included PP1, PP2A, PP2B, PP2C*β*, and PP5. Unspecific alkaline phosphatase was also tested. The CK2-inhibitor TBB used to prevent ongoing phosphorylation upon incubation with the phosphatases had no effect on the phosphatase activities as exemplified for PP2C*β* (Figure 1(b), right). 

**Figure 2:**
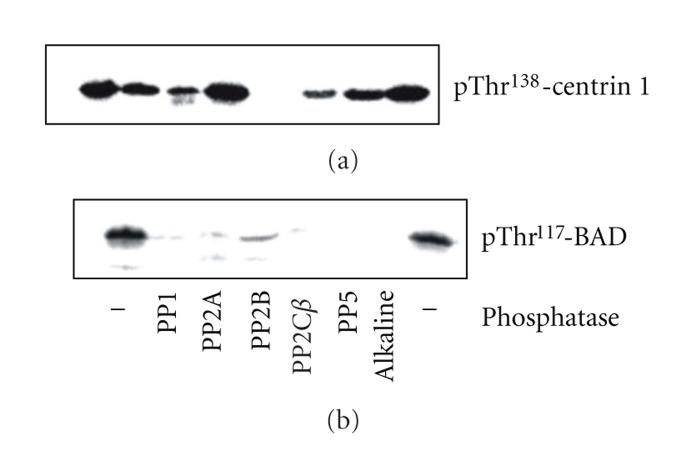
Dephosphorylation of P-centrin1 and P-BAD. (a) Incubation of P-Thr^138^-centrin1 (0.2 *μ*g) with phosphatases as indicated. (b) Incubation of P-Thr^117^-BAD (0.6 *μ*g) with phosphatases. The amount of a phosphatase added for the dephosphorylation reactions was the same in (a) and (b) (0.16 *μ*g PP1, 0.05 *μ*g PP2A, 1.3 *μ*g PP2B, 1.5 *μ*g PP2C*β*, 0.8 *μ*g PP5, or 1.5 *μ*g alkaline phosphatase). PP2C*β* is most efficient in dephosphorylating P-centrin1 phosphorylated by CK2. The BAD protein—also phosphorylated by CK2—was run for control to verify activeness of the phosphatases.

Among the 6 phosphatases tested here PP2C*β* was most efficiently dephosphorylating P-centrin1 (Figure 2(a)). All the other phosphatases tested had no or much less effect (Figure 2(a)). This unexpected selectivity prompted us to run the dephosporylation of P-BAD as an extra control. For that purpose BAD was phosphorylated at Thr^117^ by CK2 [5]. Dephosporylation of P-BAD was run in parallel and identical to the experiments dealing with the putative dephosphorylation of P-centrin1. In analogy to what is known for the majority of phosphorylation sites in any protein, our in vitro studies revealed that P-Thr^117^-BAD more or less could be hydrolyzed by all the phosphatases tested (Figure 2(b)). This was in sharp contrast to the results obtained with phosphatases acting on P-centrin1 (Figure 2(a) versus 2(b)). This unexpected result—strongest dephosphorylation of P-centrin1 by PP2C*β* (Figure 2(a))—was also observed for P-centrins2 and 4 (data not shown).

### 3.3. Characterization of Dephosphorylation of P-Centrin1 by PP2C*β*

**Figure 3:**
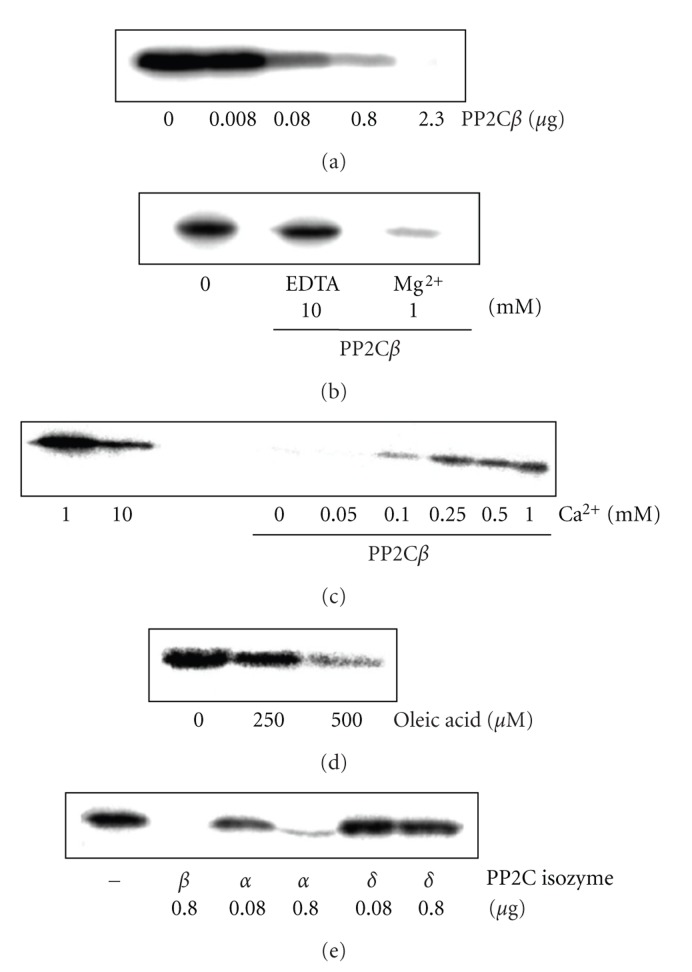
Characterization of dephosphorylation of P-centrin1 by PP2C. Centrin1 (0.2 *μ*g) was phosphorylated by CK2 (0.2 *μ*g) and [*γ* − ^32^P]ATP. (a)–(d) Dephosphorylation by 0.08 *μ*g PP2C*β* performed in the presence of 1 mM Mg^2+^ unless indicated otherwise. (a) Protein dependence. (b) Requirement for Mg^2+^-ions for activity. (c) Inhibition by Ca^2+^-ions. (d) Stimulation by oleic acid. (e) Effect of PP2C isozymes *α*, *β*, and *δ* on P-Thr^138^-centrin1.

An increasing amount of PP2C*β* protein resulted in enhanced dephosphorylation (Figure 3(a)). PP2C enzymes are characterized by their requirement for Mg^2+^- or Mn^2+^-cations for activity [6]. In line with that, dephosphorylation of P-centrin1 by PP2C*β* increased upon addition of Mg^2+^-ions (Figure 3(b)). Increasing the Ca^2+^-ion concentration reduced dephosphorylation of P-centrin1 by PP2C*β* (Figure 3(c)). Unsaturated long-chain fatty acids are inhibiting PP2C activity from plants [7] but activate PP2C*α* and PP2C*β* in vertebrates [8]. Oleic acid (18 : 1) was capable of stimulating dephosphorylation of P-centrin1 by PP2C*β* (Figure 3(d)).

Dephosphorylation of P-centrin1 was detectable not only with PP2C*β* as shown before but also with PP2C*α* (Figure 3(e)). In contrast, P-Thr^138^-centrin1 could not be hydrolyzed by PP2C*δ* (Figure 3(e)).

## 4. Discussion

Phosphorylation of centrins by CK2 occurs during dark adaptation in photoreceptor cells of the mammalian retina. It reciprocally regulates the Ca^2+^-mediated binding of centrins to the *βγ*-subunit of the visual heterotrimeric G-protein transducin [1, 9, 10]. If CK2 is constantly active in photoreceptor cilia, as seen in most systems studied so far, the identity and regulation of a phosphatase responsible for dephosphorylation of CK2-mediated centrin phosphorylation might be crucial for the biological effect of centrins.

Accordingly, in the present study, we addressed the question which phosphatase is capable of dephosphorylating P-Thr^138^-centrin1. All the most abundant retinal phosphatases were tested, that is, PP1, PP2A, PP2B, PP2C *α* and *β*, PP5, and alkaline phosphatase [11–14]. Our results were most striking: PP2C *α* and *β* most efficiently hydrolyzed P-centrin1; all other phosphatases tested had no or much less effect. This unexpected finding was verified using P-Thr^117^-BAD, phosphorylated by CK2, for control [5]. As expected, P-BAD was dephosphorylated by all those phosphatases which is in sharp contrast to the dephosphorylation of P-centrin1 by PP2C *α* and *β*.

Many proteins are phosphorylated at several distinct sites. Knowledge on the reversible phosphorylation of centrins currently comprises PKA at Ser^167^ [15–17], PKC [15], Cdc2 [15], and CK2 [18]. This report is the first focusing on phosphatases acting on P-centrins. Because of the unexpected potency of PP2C *α* and *β* to dephosphorylate CK2-mediated P-centrin1, we briefly checked whether PP2C *α* and *β* might also dephosphorylate P-centrin1 after phosphorylation by PKA. This was not the case (data not shown). Therefore, we conclude that if there is crosstalk and hierarchy among the two phosphorylation sites identified in centrin proteins, PP2C *α* and *β* are playing a most decisive role. Overall, dephosphorylation of P-centrins by PP2C *α* and *β* should increase the affinity of centrins to G_t_ *βγ* and finally reduce transport of the G-protein transducin through the connecting cilium.
